# Parallel dynamics in the yield of universal SARS-CoV-2 admission screening and population incidence

**DOI:** 10.1038/s41598-023-33824-6

**Published:** 2023-05-05

**Authors:** Peter W. Schreiber, Thomas Scheier, Aline Wolfensberger, Dirk Saleschus, Miriam Vazquez, Roger Kouyos, Walter Zingg

**Affiliations:** 1 Department of Infectious Diseases and Hospital Epidemiology, University Hospital Zurich and University of Zurich, Raemistrasse 100, 8091 Zurich, Switzerland; 2 Institute of Medical Virology, University of Zurich, Zurich, Switzerland

**Keywords:** Epidemiology, Infectious diseases

## Abstract

The majority of SARS-CoV-2 transmissions originates from either asymptomatic or presymptomatic individuals. To prevent unnoticed introduction of SARS-CoV-2, many hospitals have implemented universal admission screening during the COVID-19 pandemic. The present study aimed to investigate associations between results of an universal SARS-CoV-2 admission screening and public SARS-CoV-2 incidence. Over a study period of 44 weeks, all patients admitted to a large tertiary care hospital were tested for SARS-CoV-2 by polymerase chain reaction. SARS-CoV-2 positive patients were retrospectively categorized as symptomatic or asymptomatic at admission. Cantonal data were used to calculate weekly incidence rates per 100,000 inhabitants. We used regression models for count data to assess the association of the weekly cantonal incidence rate and the proportion of positive SARS-CoV-2 tests in the canton with (a) the proportion of SARS-CoV-2 positive individuals and (b) the proportion of asymptomatic SARS-CoV-2 infected individuals identified in universal admission screening, respectively. In a 44-week period, a total of 21,508 admission screenings were performed. SARS-CoV-2 PCR was positive in 643 (3.0%) individuals. In 97 (15.0%) individuals, the positive PCR reflected residual viral replication after recent COVID-19, 469 (72.9%) individuals had COVID-19 symptoms and 77 (12.0%) SARS-CoV-2 positive individuals were asymptomatic. Cantonal incidence correlated with the proportion of SARS-CoV-2 positive individuals [rate ratio (RR): 2.03 per 100 point increase of weekly incidence rate, 95%CI 1.92–2.14] and the proportion of asymptomatic SARS-CoV-2 positive individuals (RR: 2.40 per 100 point increase of weekly incidence rate, 95%CI 2.03–2.82). The highest correlation between dynamics in cantonal incidence and results of admission screening was observed at a lag time of one week. Similarly, the proportion of positive SARS-CoV-2 tests in the canton of Zurich correlated with the proportion of SARS-CoV-2 positive individuals (RR: 2.86 per log increase in the proportion of positive SARS-CoV-2 tests in the canton, 95%CI 2.56–3.19) and the proportion of asymptomatic SARS-CoV-2 positive individuals (RR: 6.50 per log increase in the proportion of positive SARS-CoV-2 tests in the canton, 95%CI 3.93–10.75) in admission screening. Around 0.36% of admission screenings were positive in asymptomatic patients. Admission screening results paralleled changes in population incidence with a brief lag.

## Introduction

As of May 2022, more than 518 million Coronavirus disease 2019 (COVID-19) cases and more than 6 million deaths were confirmed worldwide^1^. To slow down the spread of the causative pathogen, severe acute respiratory syndrome coronavirus 2 (SARS-CoV-2), a number of non-pharmaceutical public health measures were implemented. The measures encompassed containment strategies such as self-isolation of infected patients, identification and quarantine of exposed contacts, but also mitigation strategies such as travel restrictions, home office directives and temporary closures of shops. Increasing evidence supported SARS-CoV-2 transmission by asymptomatic or presymptomatic individuals^2–7^. Early case detection with isolation of index patients and quarantine of exposed individuals are the basics of outbreak management in general and particularly in limiting the spread of SARS-CoV-2. Escalating testing capacities were a major element early in infection control of COVID-19^8^.

Healthcare settings must protect both patients and healthcare workers from infection by SARS-CoV-2. Besides modifications in standard precautions such as introducing universal masking, many hospitals started to perform universal admission screening of patients. Data on the outcome of this strategy is limited. The objective of this study was to analyze data on universal admission screening in a large tertiary care hospital and to correlate findings with SARS-CoV-2 activity in the public.

## Material and methods

### Setting

This study was performed at the University Hospital Zurich (USZ), Zurich, Switzerland, a 941-bed tertiary-care centre featuring all medical specialties except paediatrics and orthopaedic surgery. USZ is the largest hospital in the greater Zurich area (Canton of Zurich). In 2018, approximately 18% of all hospitalized patients of the Canton were treated at USZ^9^.

### Public data

For description of the regional epidemiology during the study period, publicly available cantonal data were downloaded from the homepage of the Canton of Zurich (https://www.zh.ch/de/politik-staat/opendata.html), analysed and reported as weekly incidence rates per 100,000 inhabitants^10^. The canton of Zurich has a population of 1.56 million inhabitants^11^.

### SARS-CoV-2 testing

Data of all patients admitted between calendar week 18 of 2020 and calendar week 8 of 2021 and receiving SARS-CoV-2 testing were analysed. Throughout the study period, universal SARS-CoV-2 testing of admitted patients was mandated. Respiratory samples for SARS-CoV-2 testing were either gathered at the ward for elective admissions or at the emergency department for emergency admissions. With the exception of the Department of Obstetrics that frequently used saliva, all other departments performed nasopharyngeal swabs for SARS-CoV-2 admission testing. SARS-CoV-2 identification was done by polymerase chain reaction (PCR) testing, either using the Roche cobas SARS-CoV-2 IVD (Roche Diagnostics, Mannheim, Germany) or GeneXpert Xpress SARS-CoV-2 (Cepheid, CA, USA). Results of SARS-CoV-2 testing were usually reported within 48 h.

### Outcome definitions

All patients with a positive SARS-CoV-2 test on admission were retrospectively classified as symptomatic or asymptomatic for COVID-19 by medical chart review. “Symptomatic cases” were defined as follows^12^: respiratory symptoms and/or fever, sudden onset of anosmia or ageusia, and/or rare manifestations such as headache or diarrhoea. Medical charts were also checked for COVID-19 before admission and during the first 14 days of hospital stay. Individuals with a history of recent COVID-19 combined with resolution of symptoms and an elapsed time of at least 10 days since diagnosis and/or a Ct-value of ≥ 35 in the SARS-CoV-2 PCR were considered non-contagious and thus, treated as SARS-CoV-2 negative contributing to the denominator for calculation of rates. Primary outcome was the weekly number and proportion of asymptomatic patients with a positive SARS-CoV-2 test on admission. Further outcomes were: (1) patients with a positive SARS-CoV-2 test on admission: any new SARS-CoV-2 test on admission; (2) asymptomatic patients with COVID-19: COVID-19 without clinical symptoms, neither on admission nor during the first 14 days of hospital stay; and (3) presymptomatic COVID-19 patients: asymptomatic COVID-19 on admission with symptom development during the first 14 days of hospital stay.

### Statistical analyses

Medians with interquartile ranges (IQR) were reported for continuous variables, and absolute numbers and frequencies (%) were reported for categorical variables. The proportion of individuals with a positive test on admission was determined by dividing the number of SARS-CoV-2 positive individuals on admission by the overall number of SARS-CoV-2 testings performed on admission in corresponding calendar weeks. The proportion of asymptomatic individuals with a positive test on admission was defined as the number of asymptomatic SARS-CoV-2 positive patients on admission divided by the number of SARS-CoV-2 tests performed on admission in corresponding calendar weeks. On the cantonal level, we calculated the incidence rate per 100,000 inhabitants and calendar week, and the proportion of positive SARS-CoV-2 tests (number of SARS-CoV-2 positive individuals by the overall number of SARS-CoV-2 testings performed in corresponding calendar weeks) per calendar week. Poisson regression with offset was applied to test the association between cantonal incidence rates and the proportion of positive SARS-CoV-2 tests in the canton of Zurich with (a) the proportion of individuals with a positive test on admission and (b) the proportion of asymptomatic patients with a positive test on admission, respectively. As offset, the log of number of SARS-CoV-2 admission screening tests performed in the corresponding calendar week was coded. As sensitivity analysis, we performed negative binomial regression with the same offset. For estimating a potential lag time between the cantonal incidence rates and the results of admission screening, the cross-correlation between the time series was considered. All statistical analysis was performed using R version 4.0.3^13^. For visualization of correlations between variables for different lags assumed, lag time plots were created using the R package “astsa”^14^.

### Ethics approval

The Zurich Cantonal Ethics Commission waived the necessity for a formal ethical evaluation based on the Swiss law on research on humans (Req-2020-00441).

## Results

### Population incidence

**Figure 1:**
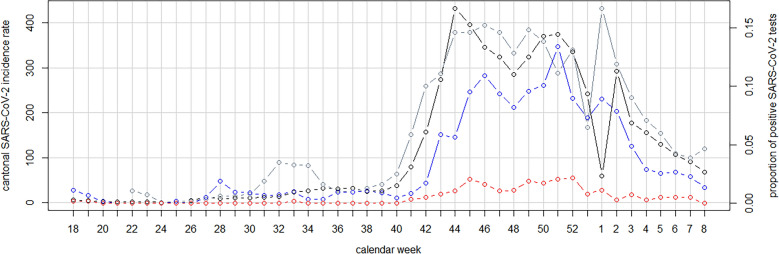
Time series of cantonal SARS-CoV-2 test results and the results of universal admission screening at the University Hospital Zurich. Black line and dots correspond to weekly cantonal incidence rate per 100,000 inhabitants. Grey line and dots correspond to the proportion of positive SARS-CoV-2 tests in the canton of Zurich (data for calendar week 18 to 21 of 2020 were not available in the cantonal dataset). Blue line and dots correspond to the proportion of patients with a positive SARS-CoV-2 test on admission per calendar week (individuals with residual SARS-CoV-2 replication after recent COVID-19 were treated as SARS-CoV-2 negative). Red line and dots correspond to the proportion of asymptomatic patients with a positive SARS-CoV-2 test on admission per calendar week.

During the study period, the median incidence rate of SARS-CoV-2 per 100,000 inhabitants and calendar week in the Canton of Zurich was 34.3 (IQR 9.9–250.2). The highest incidence rate was observed in calendar week 44/2020 with 432.1 infections per 100,000 inhabitants (Fig. 1).

### Hospital admission screening for SARS-CoV-2 at the University Hospital Zurich

In total, 21,508 patients were tested for SARS-CoV-2 on admission. In 20,865 (97.0%) patients SARS-CoV-2 PCR was negative and in 643 (3.0%) patients SARS-CoV-2 PCR was positive. Among patients with a positive SARS-CoV-2 PCR, 97 (15.0%) had a history of prior COVID-19 and were considered as non-contagious, whereas 546 (85.0%) were considered contagious. 469 (72.9%) patients showed clinical symptoms, such as cough (320, 57.7%), dyspnoea (306, 55.2%) and fever (270, 48.7%) (Supplemental Fig. 1), and 77 (12.0%) patients were asymptomatic at admission (Table 1). Per calendar week, we observed a median of 1 (IQR 0.00–2.25) asymptomatic SARS-CoV-2 positive patient on admission and a median of 6 (IQR 3.75–20.25) patients with a positive test on admission, corresponding to a median proportion of asymptomatic SARS-CoV-2 positive patients of 0.002 (IQR 0.000–0.008) and a median proportion of individuals with a positive test of 0.011 (IQR 0.006–0.0622), respectively (Fig. 1). In the follow-up of asymptomatic SARS-CoV-2 positive patients, 66 (85.7%) remained asymptomatic during the first 14 days of hospital stay.

**Table 1:** Characteristics of patients with a positive SARS-CoV-2 test on admission.

Characteristics	SARS-CoV-2 positive patients on admission	
	Any COVID-19 symptom N = 469	Asymptomatic N = 77
Age in years, median (IQR)	66 (54–74)	60 (47–72)
Gender, N (%)	Male 311 (66.3%) Female 158 (33.7%)	Male 42 (54.5%) Female 35 (45.5%)
Hospitalization due to illness of		
Internal medicine, N (%)	310 (66.1%)	18 (23.4%)
Surgical disciplines, N (%)	32 (6.8%)	38 (49.3%)
Other disciplines*, N (%)	127 (27.1%)	21 (27.3%)

*IQR* interquartile range.

*Including patients that presented at the emergency department.

### Correlation analyses of cantonal incidence rates and proportions of SARS-CoV-2 infections in admission screening

Applying Poisson regression, a significant correlation of the weekly cantonal incidence rate and both, the proportion of individuals with a positive test on admission [rate ratio (RR): 2.03 per 100 point increase of the weekly incidence rate proportion per 100,000 inhabitants, 95%CI 1.92–2.14, *P* < 0.001] and the proportion of asymptomatic patients with a positive test on admission (RR: 2.40 per 100 point increase of the weekly incidence rate per 100,000 inhabitants, 95%CI 2.03–2.82, *P* < 0.001) was identified. These associations were confirmed in sensitivity analyses using negative binomial regression (proportion positive: RR 2.21 per 100 point increase of the weekly incidence rate per 100,000 inhabitants, 95%CI 1.96–2.49, *P* < 0.001; proportion of asymptomatic patients with a positive SARS-CoV-2 test on admission: RR 2.44 per 100 point increase of the weekly incidence rate per 100,000 inhabitants, 95%CI 2.05–2.89, *P* < 0.001).

**Figure 2:**
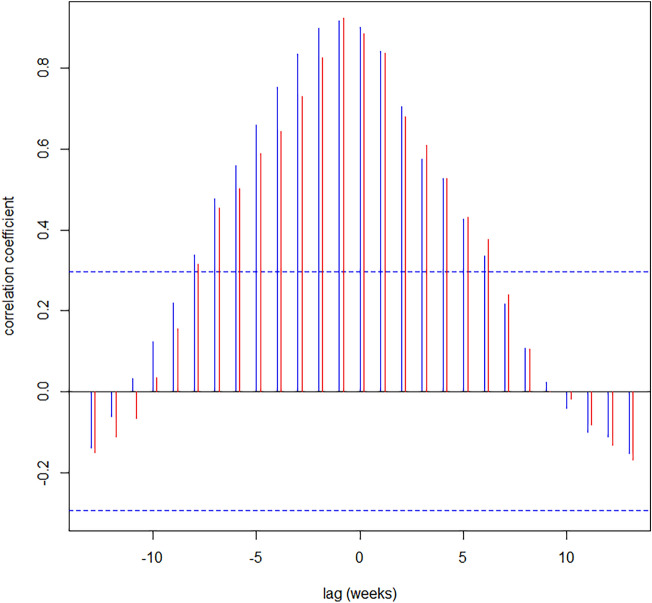
Cross-correlation of cantonal incidence rate and the proportion of individuals with a positive SARS-CoV-2 test on admission (vertical blue lines) and the proportion of asymptomatic patients with a positive SARS-CoV-2 test on admission (vertical red lines). x-axis: lag in calendar weeks. y-axis: correlation coefficient per given lag time.

Visual inspection of the time series plots of the cantonal incidence rate and (a) the proportion of individuals with a positive test on admission and (b) the proportion of asymptomatic patients with a positive test on admission indicated parallel dynamics with a lag time (Fig. 1). To identify a potential delay between changes in cantonal incidence and the results of admission screening, we additionally performed cross-correlation analyses (Fig. 2). The highest correlation coefficient was observed for a lag time of 1 calendar week for both, the proportion positive and the proportion of asymptomatic patients with a positive SARS-CoV-2 test on admission (Supplemental Figs. 2, 3). However, the correlation was also very high without assuming a lag between the time series (proportion positive: rho = 0.90 for lag of 0 weeks vs. 0.92 for a lag of 1 week; proportion of asymptomatic patients with a positive SARS-CoV-2 test on admission: rho = 0.89 for lag of 0 weeks vs. 0.93 for a lag of 1 week).

### Correlation analyses of the proportion of positive SARS-CoV-2 tests in the canton and proportions of SARS-CoV-2 infections in admission screening

In Poisson regression, a significant association of the proportion of positive SARS-CoV-2 tests in the canton and both, the proportion of individuals with a positive test on admission (RR: 2.86 per log increase in the proportion of positive SARS-CoV-2 tests in the canton, 95%CI 2.56–3.19, *P* < 0.001) and the proportion of asymptomatic patients with a positive test on admission (RR: 6.50 per log increase in the proportion of positive SARS-CoV-2 tests in the canton, 95%CI 3.93–10.75, *P* < 0.001) was detected (Fig. 1). Sensitivity analyses with negative binomial regression confirmed these associations (proportion positive: RR 2.49 per log increase in the proportion of positive SARS-CoV-2 tests in the canton, 95%CI 2.08–2.98, *P* < 0.001; proportion of asymptomatic patients with a positive SARS-CoV-2 test on admission: RR 6.50 per log increase in the proportion of positive SARS-CoV-2 tests in the canton, 95%CI 3.93–10.75, *P* < 0.001).

## Discussion

In the present study on universal SARS-CoV-2 admission screening encompassing 44 weeks and more than 20,000 patients, around 3% of patients undergoing a SARS-CoV-2 PCR on hospital admission tested positive of which 12% were asymptomatic. The rate of asymptomatic patients with a positive SARS-CoV-2 test on admission paralleled, but slightly lagged the population incidence.

The proportion of asymptomatic patients with a positive SARS-CoV-2 test on admission (0.36%) is similar to previous findings in our hospital^15^ but higher compared to reports from other hospitals in Switzerland (0.15%)^16^ or abroad (0.005% and 0.16%)^17,18^. Most asymptomatic SARS-CoV-2 positive patients on admission did not develop COVID-19 symptoms during hospital stay; this finding resembles a recent report from another Swiss tertiary care centre where one out of six asymptomatic SARS-CoV-2-positive patients became symptomatic over the course of the hospitalization^16^.

In contrast to the studies of Stadler et al.^16^ and Jung et al.^17^, we identified a significant correlation of the population incidence with both, the proportion of individuals with a positive test on admission and the proportion of asymptomatic patients with a positive test on admission. In line with our findings, Krüger et al. reported variation in the proportion of individuals with a positive test on admission screening by population incidence^18^. The authors divided an overall observation period of 4 weeks in high (before peak incidence in Bavaria during observation period) and low (after peak incidence in Bavaria during observation period) incidence periods and compared the proportions. Taking the dynamics of the pandemic better into account, we addressed the correlation between cantonal incidence and results of admission screening on a weekly basis.

For the period from calendar week 22 in 2020 until to calendar week 8 in 2021, the cantonal dataset also included information on the proportion of positive SARS-CoV-2 tests performed in the canton of Zurich. In time series analysis plots, the dynamics in the proportion of positive SARS-CoV-2 tests on the cantonal level paralleled the cantonal incidence rate. This finding is similar to the results of Nesteruk^19^. In line with the findings for the cantonal incidence rate, significant associations of the proportion of positive SARS-CoV-2 tests performed in the canton and (a) the proportion of individuals with a positive SARS-CoV-2 test and (b) the proportion of asymptomatic SARS-CoV-2 infected individuals identified in universal admission screening were detected. The parallel increase in the incidence rate and the proportion of positive SARS-CoV-2 tests on the cantonal level reflects insufficient testing during periods with high SARS-CoV-2 activity.

Following the exponential increase in population incidence, a one-week lag time was observed until a parallel dynamic in the proportion positive of admission screening was detected, regardless of having symptoms or being asymptomatic. One hypothesis for this lag time could be that the initial phase of the exponential incidence growth in the population is driven by younger age groups. Among hospitalized patients in Switzerland, older age is common with 36.9% of patients ≥ 65y in 2019^20^. Recent reports indicated an increase in the proportion of younger individuals among all SARS-CoV-2 infections^21^. Even if younger individuals are less likely to suffer from severe COVID-19 resulting in hospitalization, some might transmit SARS-CoV-2 to elderly individuals. US data indicated a preceding increase in the percentage of positive SARS-CoV-2 test results among younger adults prior to an increase in elderly individuals^22^. If an intermittent universal admission screening is considered, one strategy could be an introduction immediately after a rapid rise in population incidence. Future studies confirming our observation of a certain delay until an increase of SARS-CoV-2 in the community is associated with a higher proportion of a- or presymptomatic SARS-CoV-2 individuals at hospital admission, seem desirable, to support the proposed strategy.

Throughout the study period, mainly SARS-CoV-2 wild type and later alpha variant predominated in Switzerland^23^. Vaccination started at the end of 2020 focussing initially on vulnerable populations. At the end of the study period vaccination coverage was still low with 9.47 vaccinated individuals per 100,000 inhabitants^24^. The applicability of our findings for settings with predominance of other SARS-CoV-2 variants and much higher vaccination coverage remains unsure and future studies seem warranted.

The present study has several strengths. We evaluated data collected over a time span of 44 weeks encompassing more than 20,000 admitted patients. The vast majority of prior studies on this research question were characterized by a shorter duration^15–18^ or limited sample size^15,16,18^. The availability of corresponding longitudinal data on the cantonal level enabled a comparison of cantonal incidence with the proportion of individuals with a positive test on admission screening irrespective of symptoms of COVID-19. We were able to identify non-contagious individuals with prior COVID-19 and residual viral replication; this allowed us to focus the analysis on contagious individuals. In addition, medical chart review of individuals with a positive test on admission but without symptoms of COVID-19 enabled us to assess potential contagiousness during hospital stay and to distinguish between a- and presymptomatic infection.

The study also has several limitations. It was a single centre study and the classification of symptomatic or asymptomatic at admission was performed retrospectively. However, as our hospital participated in an antecedent study with prospective collection of COVID-19 symptoms at admission^15^, we have confidence that documentation in patient charts has improved following this intervention. In addition, the method we used for the correlation analysis between admission screening and cantonal incidence data assumes independence between data points, which is not the case. The degree of evidence resulting from this approach is likely overestimated. However, independently of the statistical significance of the associations, we found very high correlations (rho > 0.90) arguing that this effect is relevant. One specific limitation of the Poisson regression is that it does not allow for overdispersion. To address this limitation, we added negative binomial regression models that confirmed the findings. The use of saliva samples in the Department of Obstetrics may have resulted in some underestimation of infectiousness among SARS-CoV-2 positive individuals. A prior study with participation of our centre comparing SARS-CoV-2 detection in saliva and nasopharyngeal swabs supported saliva as a generally reliable specimen for SARS-CoV-2 detection, but average Ct values were lower in saliva samples compared to nasopharyngeal swabs^25^. No information on the frequency of symptoms of individuals tested in the Canton of Zurich was available, thus hindering an analysis of a correlation with results of universal admission screening.

Future studies taking secondary attack rates of a- and presymptomatic SARS-CoV-2 infected individuals for benefit assessment into account, seem desirable. Notably, a recent meta-analysis supported lower secondary attack rates from asymptomatic individuals compared to presymptomatic or symptomatic individuals^26^.

## Conclusions

The proportion of SARS-CoV-2 positive individuals at hospital admission as well as the proportion of asymptomatic SARS-CoV-2 infected individuals identified in universal admission screening paralleled dynamics in population incidence. The highest correlation was found at a lag time of 1 week. Resembling to the findings for population incidence, the proportion of positive SARS-CoV-2 tests in the canton correlated with the results of universal admission screening. Especially during periods of high population incidence, universal admission screening could contribute to prevention of nosocomial SARS-CoV-2 infections.

## Supplementary Information

Supplementary Information.

## Data Availability

The datasets used and/or analysed during the current study are available from the corresponding author on reasonable request.
