# Supplementary material for: Parallel dynamics in the yield of universal SARS-CoV-2 admission screening and population incidence
Source: Sci Rep. 2023 May 5;13:7296. doi: 10.1038/s41598-023-33824-6 (PMC10160732; doi:10.1038/s41598-023-33824-6)
Supplement: Supplementary file 1 — Supplementary Information. [file 41598_2023_33824_MOESM1_ESM.docx]

**Supplemental Figures**


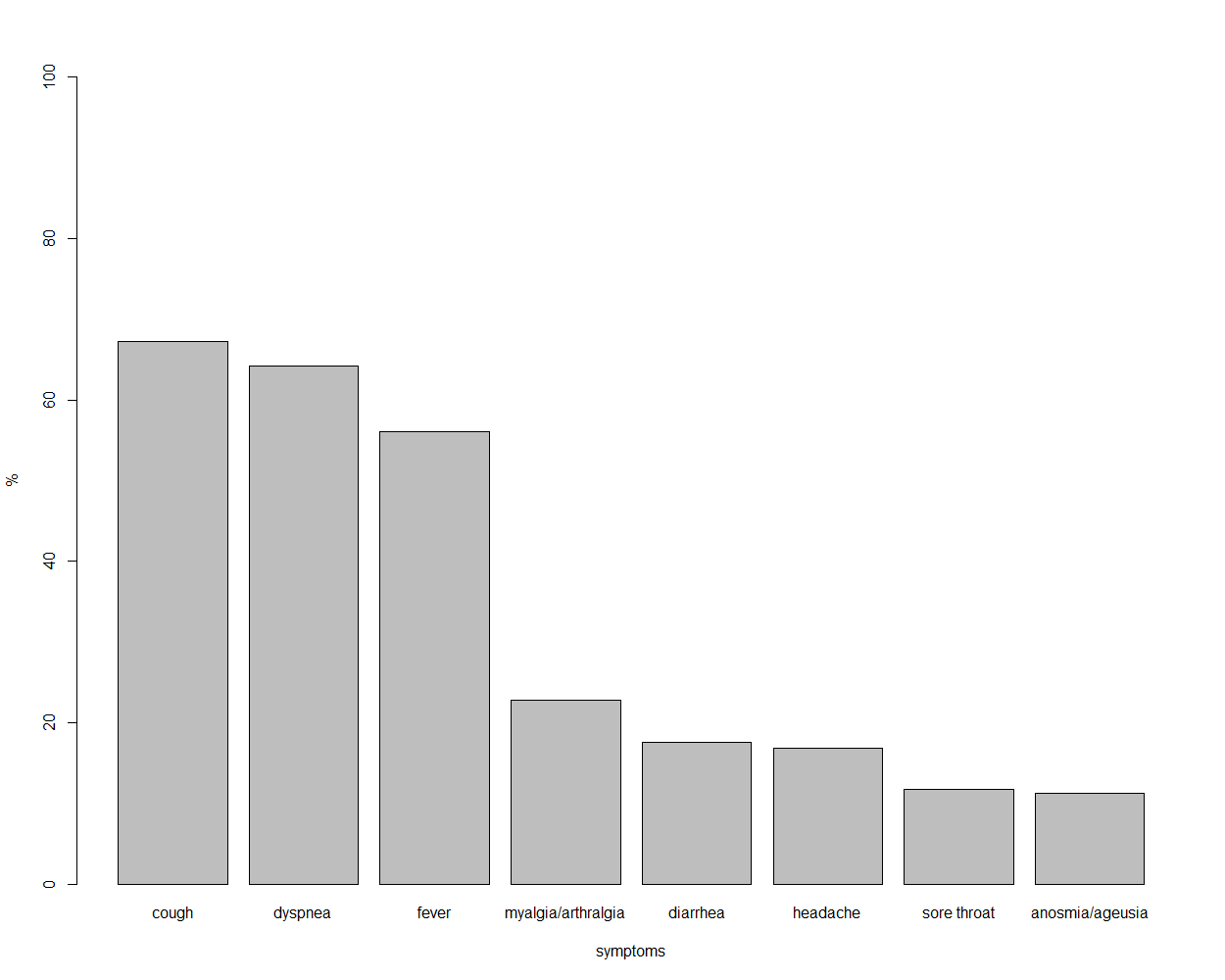


**Supplemental Figure 1:** COVID-19 symptoms among 469 symptomatic SARS-CoV-2 infected patients


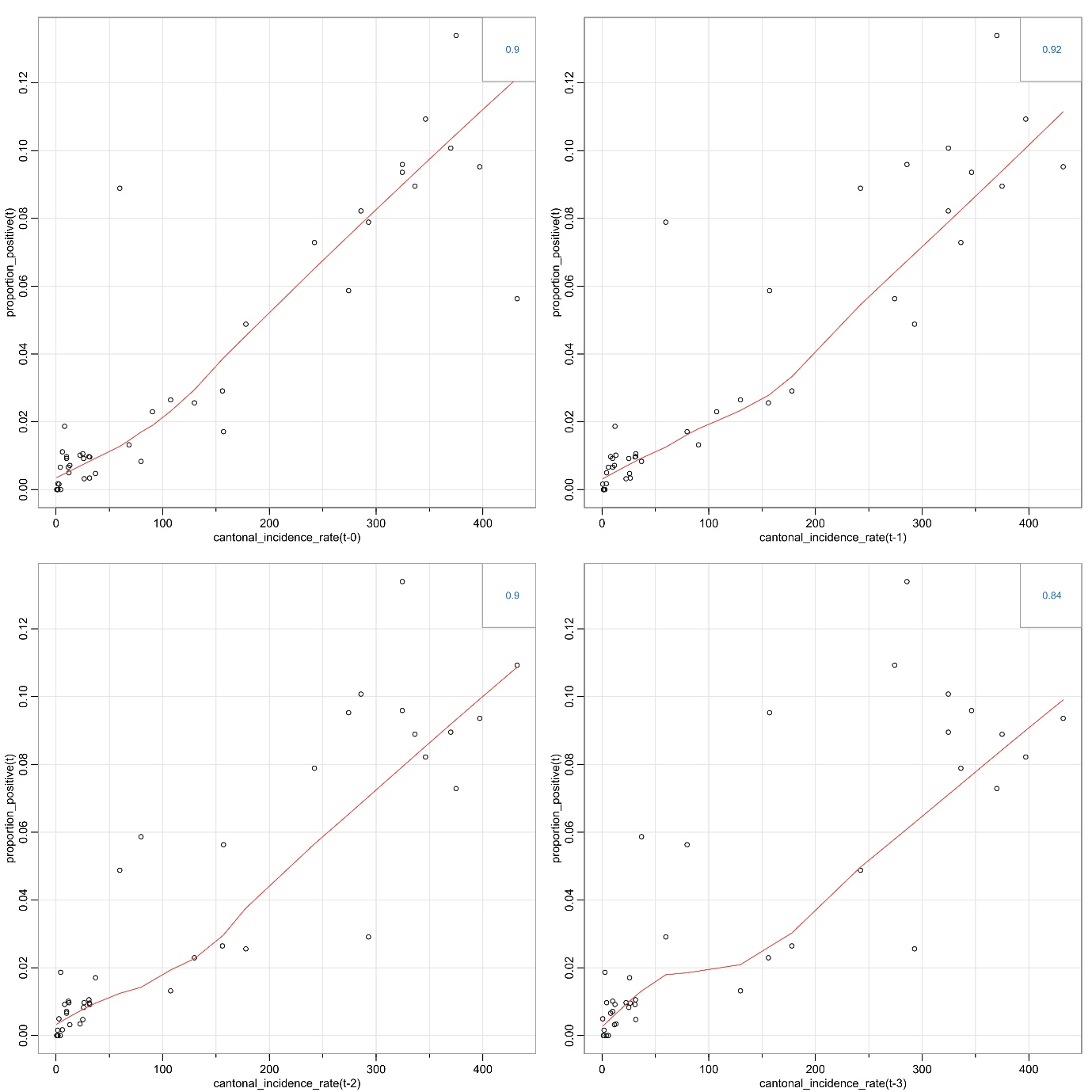


**Supplemental Figure 2:** Lag plot for correlation of cantonal SARS-CoV-2 incidence rate and proportion of individuals with a positive SARS-CoV-2 test on admission


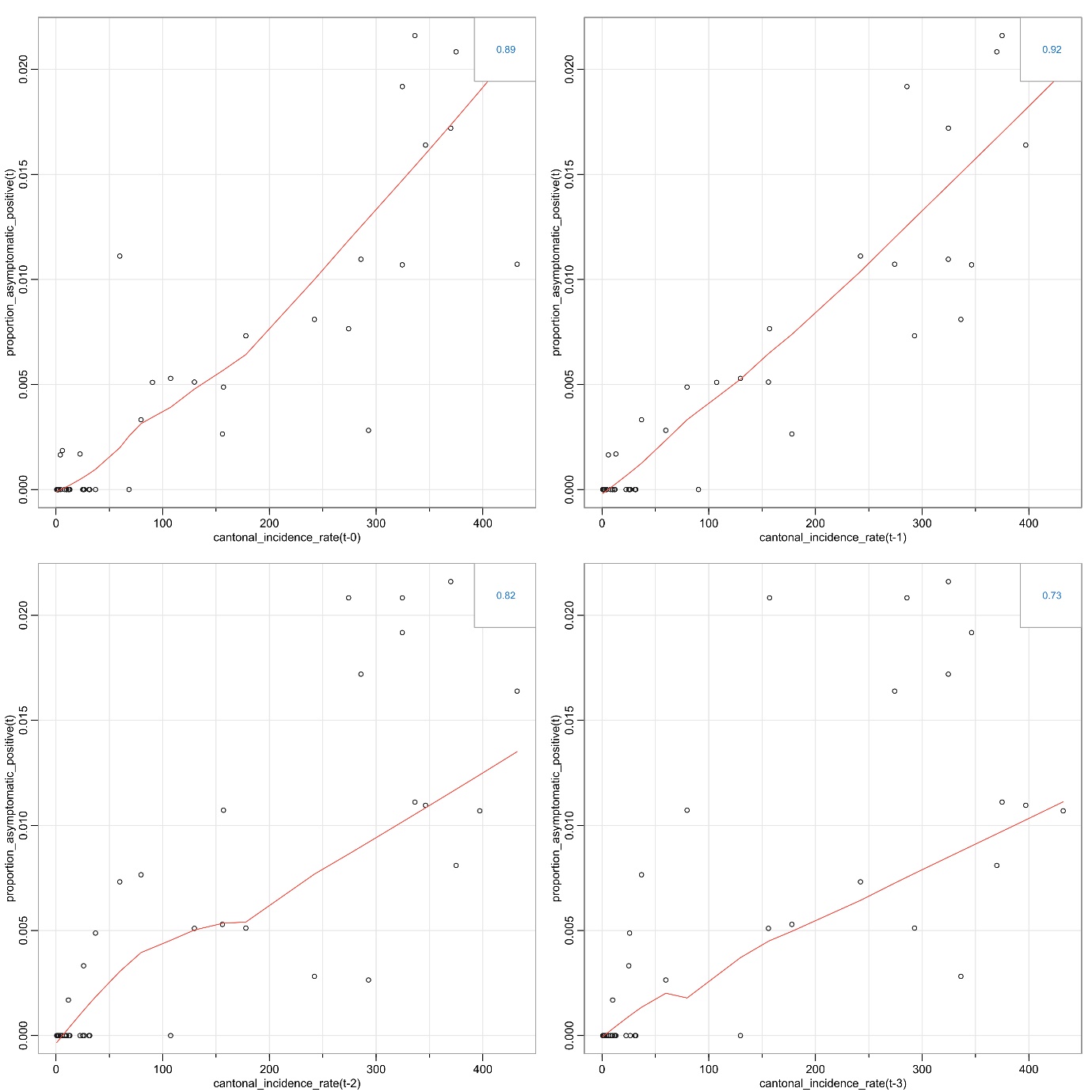


**Supplemental Figure 3:** Lag plot for correlation of cantonal SARS-CoV-2 incidence rate and the proportion of asymptomatic patients with a positive SARS-CoV-2 test on admission
